# Supplementary material for: Sex-specific DNA methylation associations with circulating urate levels and BCG-induced urate changes
Source: Commun Med (Lond). 2025 Jul 31;5:321. doi: 10.1038/s43856-025-01044-w (PMC12313909; doi:10.1038/s43856-025-01044-w)
Supplement: Supplementary file 2 — Supplementary Information [file 43856_2025_1044_MOESM2_ESM.pdf]

Supplementary Figure 1

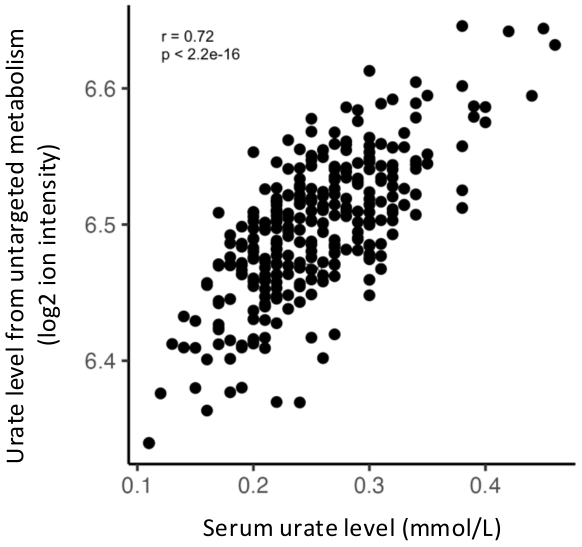

Supplementary Figure 1. Scatter plot showing the strong correlation between two methods for circulating urate measurement.

Supplementary Figure 2

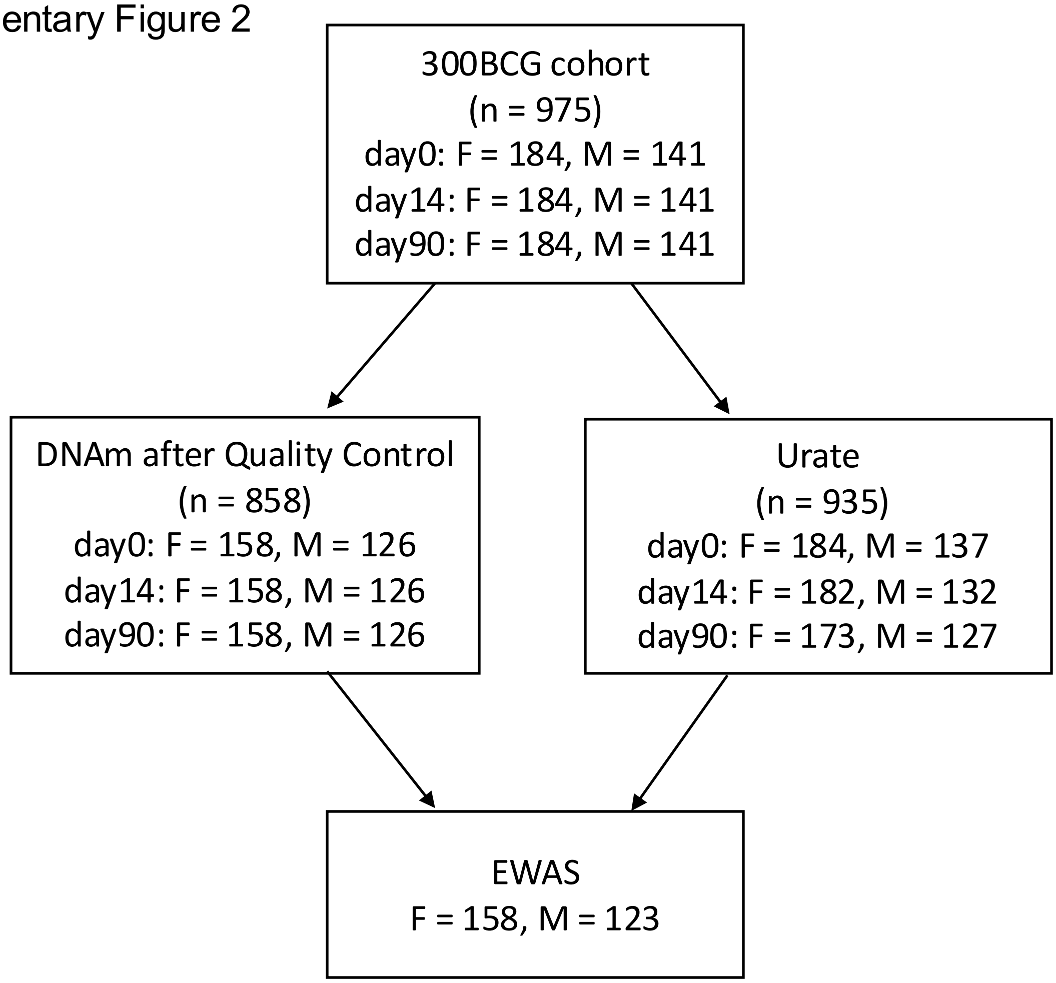

Supplementary Figure 2. Flow diagram of the number of samples collected in the discovery study.

Supplementary Figure 3

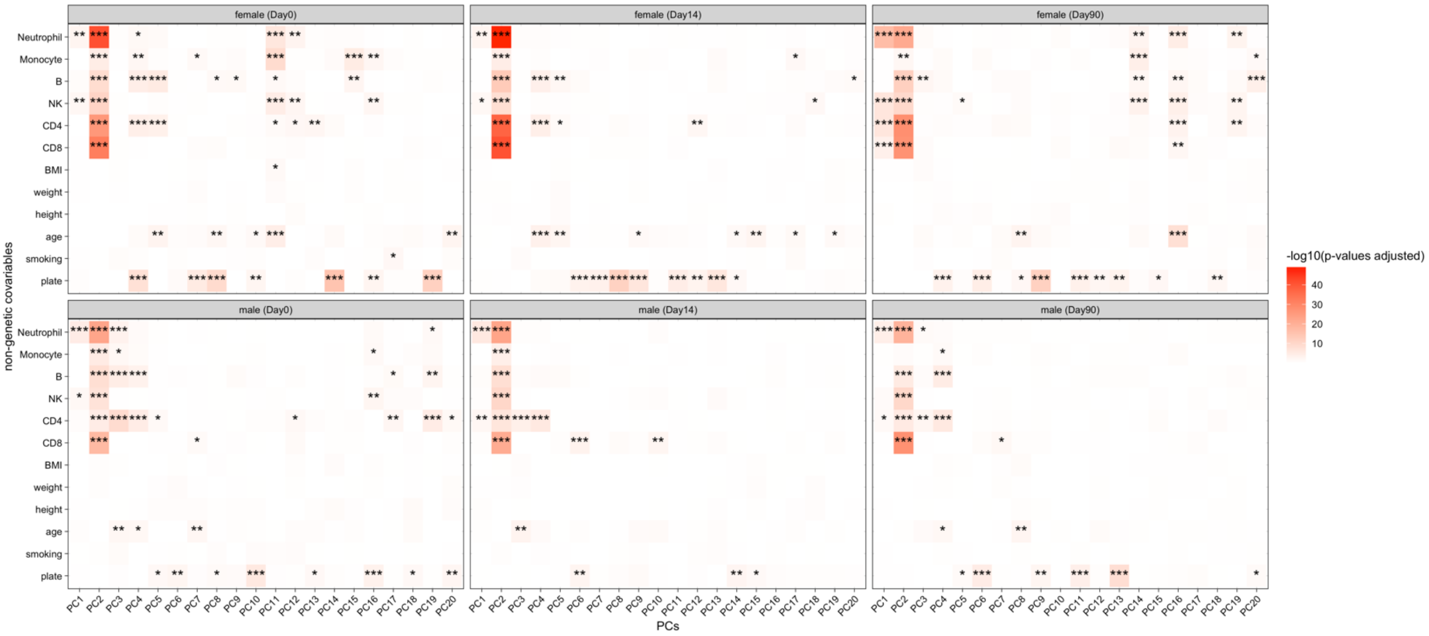

Supplementary Figure 3. Cell proportions, batch (sample plate), and age were significantly associated with the top PCs calculated from DNAm. The spearman method was used to calculate the correlation between the continuous variables with the top PCs. Kruskal-Wallis test by rank was used for the discrete variable. *: P < 0.05, **: P < 0.01, ***: P < 0.005, ****: P < 0.001.

Supplementary Figure 4

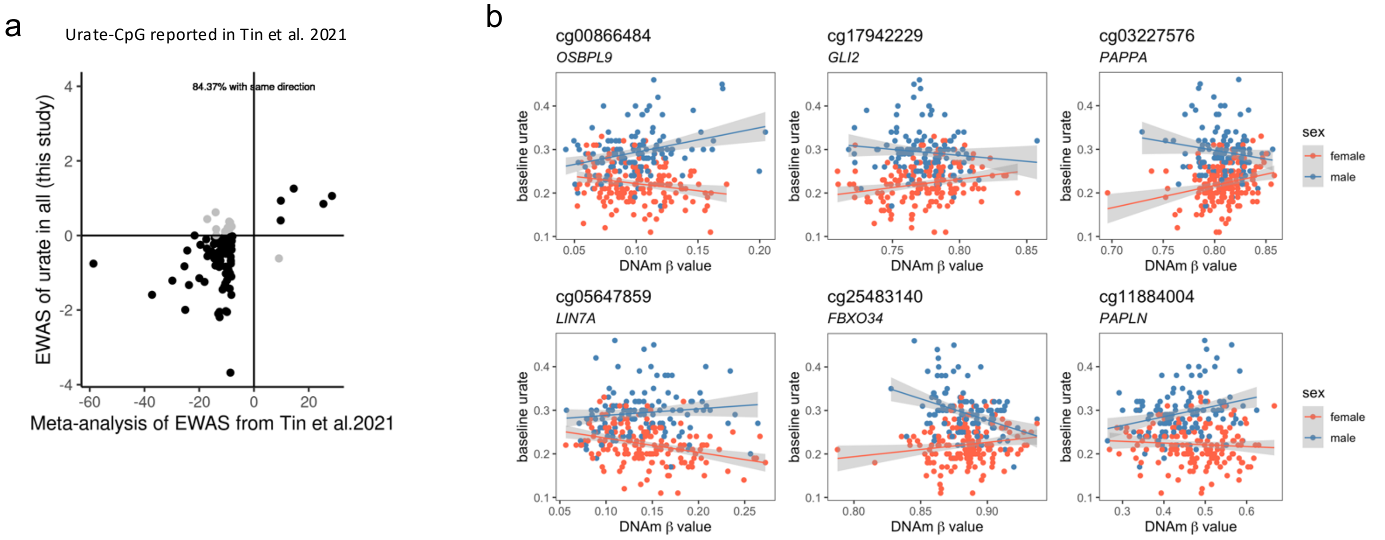

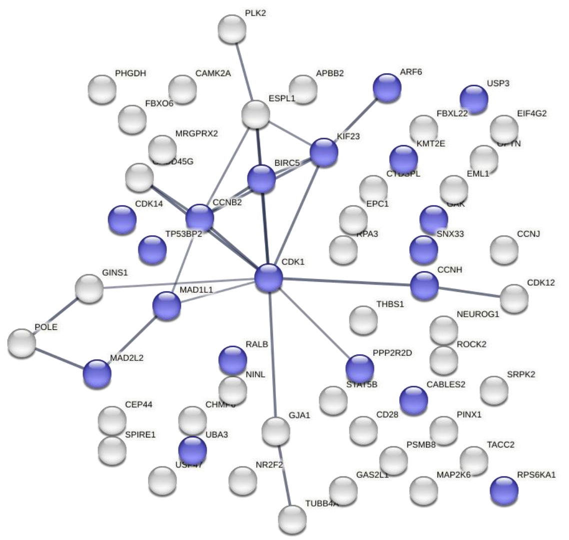

Functional enrichments in the protein-protein interaction network
purple dot: cell cycle, FDR = 0.0006

d

Rank	Motif	Name	P-value	log P-pvalue	q-value (Benjamini)
1	GAAAGTGAAAGT	IRF1(IRF)/PBMC-IRF1-ChIP-Seq(GSE43036)/Homer	1e-2	-6.623e+00	0.5850
2	CTTGTTTACATA	Foxa2(Forkhead)/Liver-Foxa2-ChIP-Seq(GSE25694)/Homer	1e-2	-6.602e+00	0.5850
3	GATGACGTCA	Atf1(bZIP)/K562-ATF1-ChIP-Seq(GSE31477)/Homer	1e-2	-6.482e+00	0.5850
4	CACTTCCGGT	Fli1(ETS)/CD8-FLI-ChIP-Seq(GSE20898)/Homer	1e-2	-5.881e+00	0.5850
5	GGCAATGAAA	Hoxd10(Homeobox)/ChickenMSG-Hoxd10.Flag-ChIP-Seq(GSE86088)/Homer	1e-2	-5.832e+00	0.5850
6	CAGGGCTGTGTAAACAGA	Fox:Ebox(Forkhead,bHLH)/Panc1-Foxa2-ChIP-Seq(GSE47459)/Homer	1e-2	-5.389e+00	0.5850
7	CCTTGTTTACATAGG	Foxa3(Forkhead)/Liver-Foxa3-ChIP-Seq(GSE77670)/Homer	1e-2	-5.316e+00	0.5850
8	ACTGAAACCA	IRF4(IRF)/GM12878-IRF4-ChIP-Seq(GSE32465)/Homer	1e-2	-5.291e+00	0.5850
9	GGCACTCAAG	Nkx2.1(Homeobox)/LungAC-Nkx2.1-ChIP-Seq(GSE43252)/Homer	1e-2	-5.262e+00	0.5850
10	GGAAGTGAAAGT	IRF8(IRF)/BMDM-IRF8-ChIP-Seq(GSE77884)/Homer	1e-2	-5.164e+00	0.5850

e

Rank	Motif	Name	P-value	log P-pvalue	q-value (Benjamini)
1	TTCAAAATAAAAGTC	MYNN(Zf)/HEK293-MYNN.eGFP-ChIP-Seq(Encode)/Homer	1e-2	-5.474e+00	1.0000
2	TTGAGTGGTT	Bapx1(Homeobox)/VertebralCol-Bapx1-ChIP-Seq(GSE36672)/Homer	1e-2	-5.151e+00	1.0000
3	AACCACTCAA	Nkx2.5(Homeobox)/HL1-Nkx2.5.biotin-ChIP-Seq(GSE21529)/Homer	1e-2	-4.877e+00	1.0000
4	ACCGGAAGTG	ETV4(ETS)/HepG2-ETV4-ChIP-Seq(ENCODE)/Homer	1e-2	-4.844e+00	1.0000
5	AAGCACTTAA	Nkx3.1(Homeobox)/LNCaP-Nkx3.1-ChIP-Seq(GSE28264)/Homer	1e-2	-4.814e+00	1.0000

Supplementary Figure 4. Interaction analyses identified 6 CpG sites that were associated with urate level at baseline in a sex-dependent manner. (a) Each dot represented urate-associated CpG sites reported from a large meta-analysis of EWAS from Tin et al. Scatter plot showing the distribution of -log10(P value) * sign (effect size) of these reported urate-associated CpG sites in Tin et al. and BCG cohort. Black dots represented CpG sites with the same effect direction, while grey dots represent CpG sites with different effect direction. (b) Scatter plot showing the correlation between $urate_{baseline}$ and $DNAm_{baseline}$ β value in females and males separately. (c) Protein-protein interaction networks functional enrichment analysis of the urate-associated CpG sites from females. (d) Top 10 enriched motifs for urate-associated CpG sites identified from males. (e) Top enriched motifs for urate-associated CpG sites identified from females.

Supplementary Figure 5

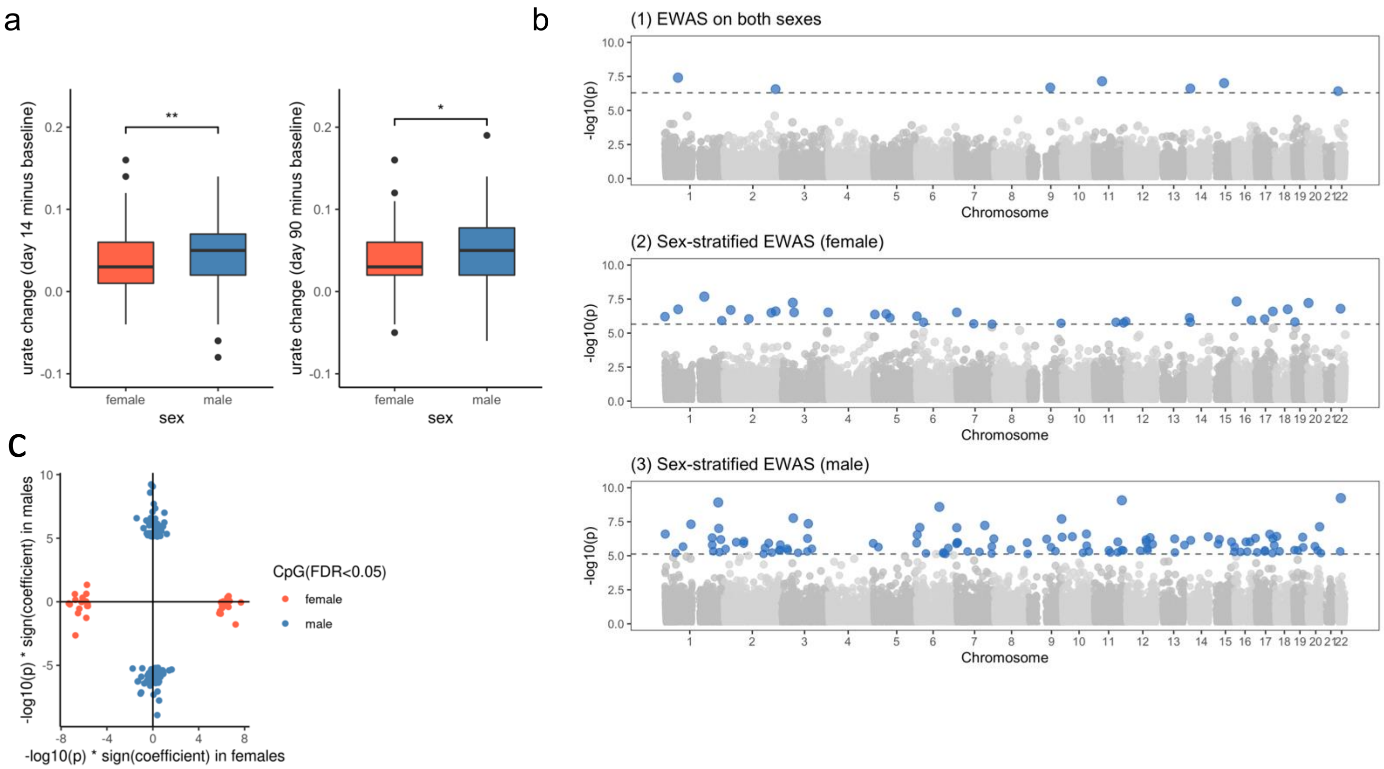

Supplementary Figure 5. $\Delta Urate_{long}$(day90-day0) was associated with $DNAm_{baseline}$ in a sex-specific manner. (a) Boxplot showing the $\Delta Urate_{short}$ and $\Delta Urate_{long}$ in females and males, color indicating different sex groups. One-sided Wilcoxon rank sum test was used to compare the difference between males and females. *: $P < 0.05$, **: $P < 0.01$. (b) Manhattan plots showing the results from EWAS of $\Delta urate_{long}$ in (1) all participants, (2) females, and (3) males respectively. The CpG sites are ordered by their chromosomal position on the x-axis with their -log10(P value) of the association on the y-axis, with dotted horizontal lines indicating the level of significance corrected for multiple testing (FDR < 0.05). Blue dots represent significant CpG sites and grey dots represent non-significant CpG sites. (c) The scatter plot showing the distribution of -log10(P value) * sign(effect size) of the significant sites from EWAS of $\Delta urate_{long}$ in males and females. The color of the dots represents the significant sites from different sex groups.

Supplementary Figure 6

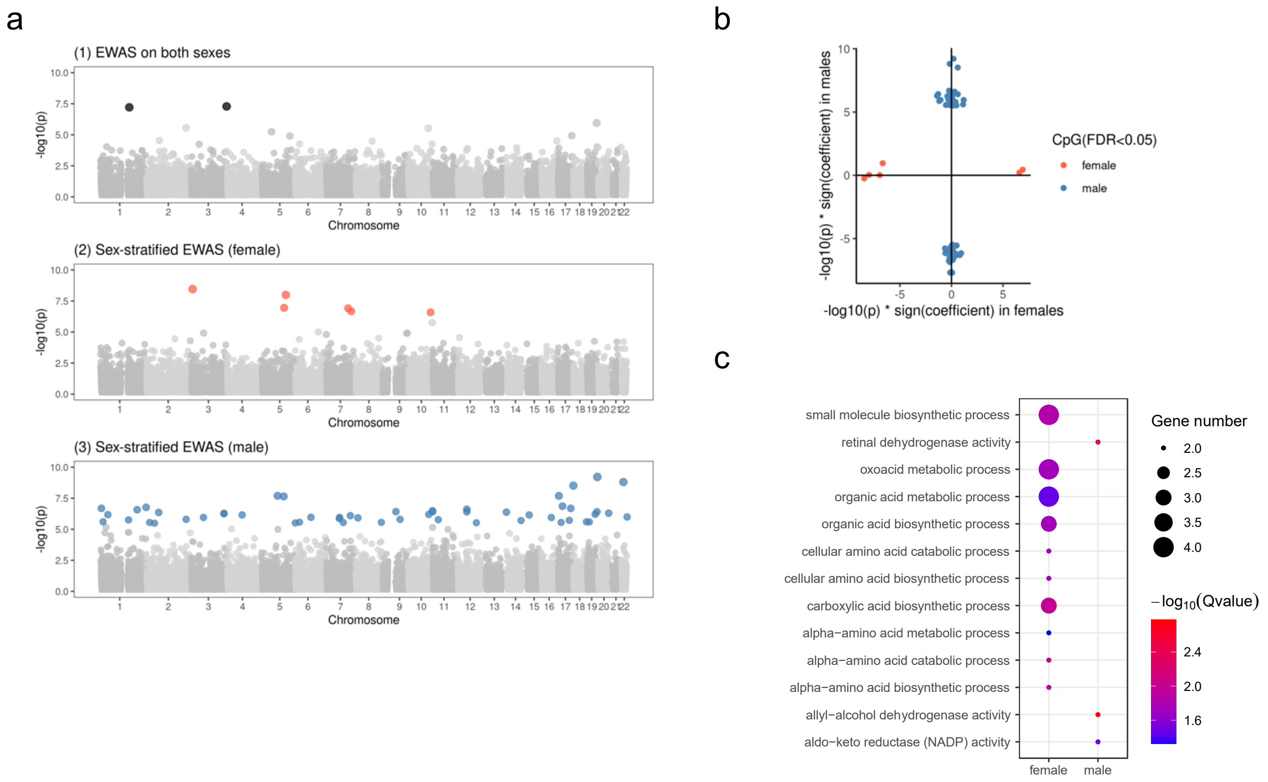

Supplementary Figure 6. Δurate(day90-day0) was associated with ΔDNAm(day90-day0) upon BCG vaccination in a sex-specific manner. (a) The Manhattan plot showing the results from the epigenome-wide association between $\Delta Urate_{long}$ and $\Delta DNAm_{long}$ in all participants (1), females (2), and males (3) respectively. The CpG sites were ordered by their chromosomal position on the x-axis with their -log10(P value) of the association on the y-axis. Colorful dot: significant CpG sites, grey dot: non-significant CpG sites. (b) The scatter plot showing the distribution of -log10(P value) * sign(effect estimate) of the significant sites from the epigenome-wide association between $\Delta Urate_{long}$ and $\Delta DNAm_{long}$ in males and females. The color represented the significant sites from different sex groups. (c) Dot plot describing the KEGG enrichment categories of genes annotated to the significant CpG sites identified from the epigenome-wide association between $\Delta Urate_{long}$ and $\Delta DNAm_{long}$. The color and size of the dot indicated the significance and the number of annotated genes in each category.

Supplementary Figure 7

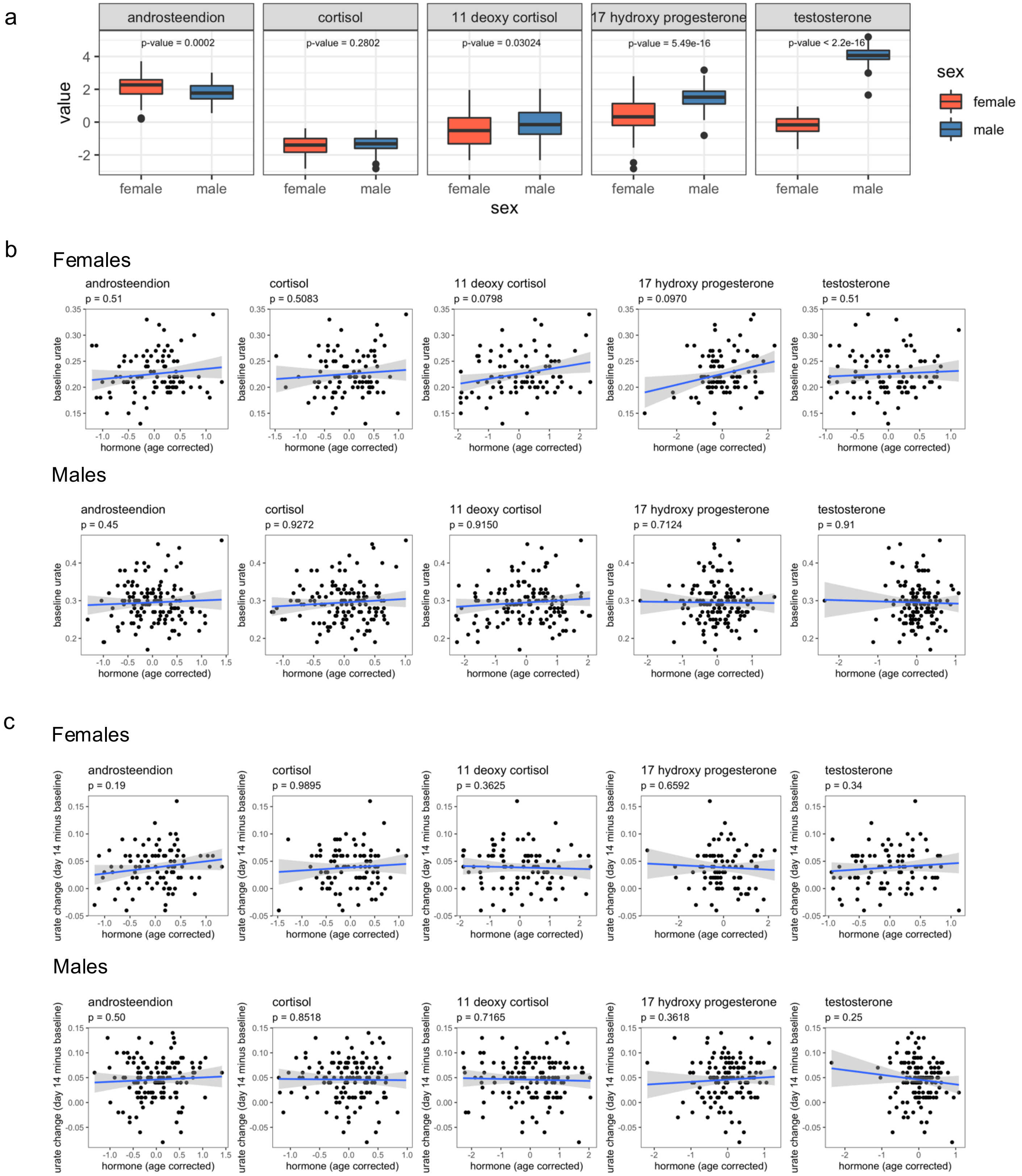

Supplementary Figure 7. Baseline hormones were not correlated with $urate_{baseline}$ and $\Delta Urate_{short}$. (a) Hormone levels in males and females. (b) Spearman's correlation between $urate_{baseline}$ and baseline hormones (after age correction). (c) Spearman's correlation between $\Delta Urate_{short}$ and baseline hormone (after age correction).

Supplementary Figure 8

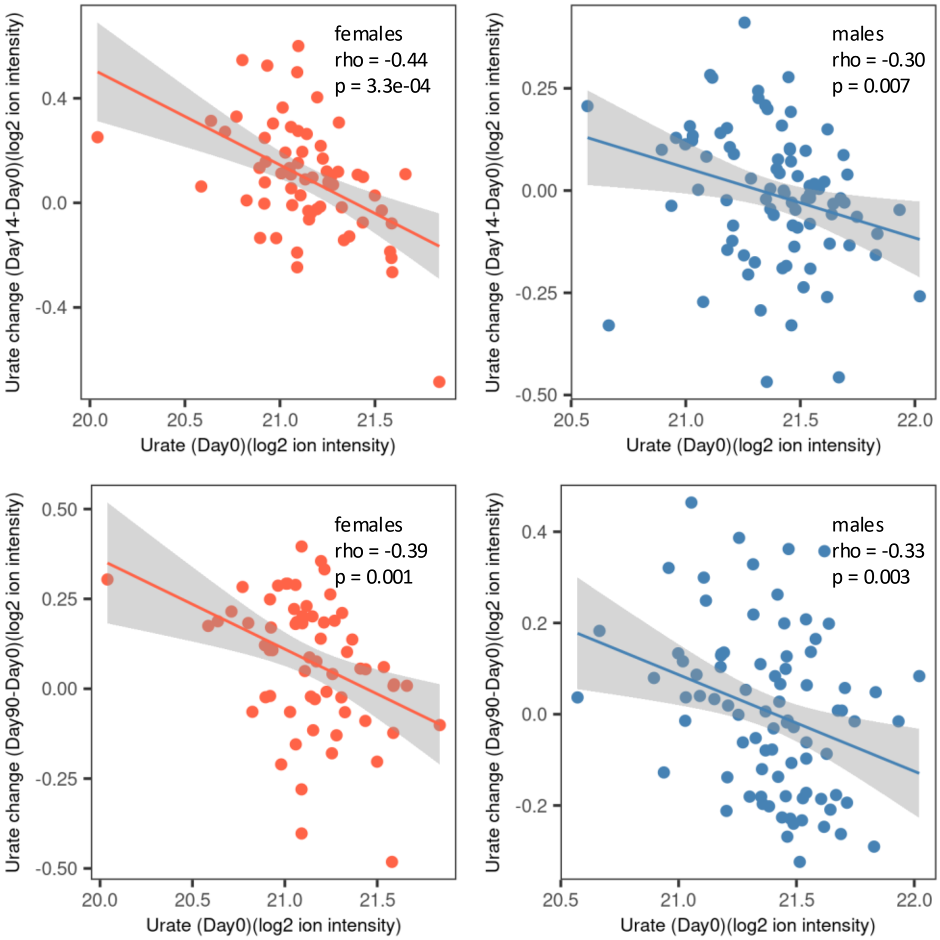

Supplementary Figure 8. Spearman's correlation between ΔUrate and $Urate_{baseline}$. The color indicated different sex groups. (red: females, blue: males)
