# Supplementary material for: Sex-specific DNA methylation associations with circulating urate levels and BCG-induced urate changes
Source: Commun Med (Lond). 2025 Jul 31;5:321. doi: 10.1038/s43856-025-01044-w (PMC12313909; doi:10.1038/s43856-025-01044-w)
Supplement: Supplementary file 3 — Description of Additional Supplementary Files [file 43856_2025_1044_MOESM3_ESM.pdf]

Description of additional supplementary file

File name: Supplementary Data 1

Description: Basic information of the study cohort

File name: Supplementary Data 2

Description: Baseline urate-associated CpG in males that reached epigenome-wide significance with FDR adjusted P below 0.05

File name: Supplementary Data 3

Description: Baseline urate-associated CpG in females that reached epigenome-wide significance with FDR adjusted P below 0.05

File name: Supplementary Data 4

Description: eQTM of the urate-associated CpG sites identified in males

File name: Supplementary Data 5

Description: Urate-associated CpG sites (from males) in discovery cohort and replication cohorts

File name: Supplementary Data 6

Description: Urate change(Day14-Day0)-associated CpG in males that reached epigenome-wide significance with FDR adjusted P below 0.05

File name: Supplementary Data 7

Description: Urate change(Day14-Day0)-associated CpG in females that reached epigenome-wide significance with FDR adjusted P below 0.05

File name: Supplementary Data 8

Description: Enrichment of urate change-associated CpG from females

File name: Supplementary Data 9

Description: Enrichment of urate change-associated CpG from males

File name: Supplementary Data 10

Description: Urate change(Day90-Day0)-associated CpG in males that reached epigenome-wide significance with FDR adjusted P below 0.05

File name: Supplementary Data 11

Description: Urate change(Day90-Day0)-associated CpG in females that reached epigenome-wide significance with FDR adjusted P below 0.05

File name: Supplementary Data 12

Description: Significant associations between urate change(Day14-Day0) and DNA methytlation change in males that reached epigenome-wide significance with FDR adjusted P below 0.05

File name: Supplementary Data 13

Description: Significant associations between urate change(Day14-Day0) and DNA methytlation change in females that reached epigenome-wide significance with FDR adjusted P below 0.05

File name: Supplementary Data 14

Description: Significant associations between urate change(Day90-Day0) and DNA methytlation change in males that reached epigenome-wide significance with FDR adjusted P below 0.05

File name: Supplementary Data 15

Description: Significant associations between urate change(Day90-Day0) and DNA methytlation change in females that reached epigenome-wide significance with FDR adjusted P below 0.05

File name: Supplementary Data 16

Description: X-chromosomal-wide association of urate or urate change
