# Supplementary material for: Sex-specific DNA methylation associations with circulating urate levels and BCG-induced urate changes
Source: Commun Med (Lond). 2025 Jul 31;5:321. doi: 10.1038/s43856-025-01044-w (PMC12313909; doi:10.1038/s43856-025-01044-w)
Supplement: Supplementary file 20 — Reporting Summary [file 43856_2025_1044_MOESM20_ESM.pdf]

nature portfolio

Corresponding author(s): Dr. Cheng-Jian Xu

Last updated by author(s): 19/06/25

Reporting Summary

Nature Portfolio wishes to improve the reproducibility of the work that we publish. This form provides structure for consistency and transparency in reporting. For further information on Nature Portfolio policies, see our Editorial Policies and the Editorial Policy Checklist.

Statistics

For all statistical analyses, confirm that the following items are present in the figure legend, table legend, main text, or Methods section.

n/a	Confirmed	
☐	☒	The exact sample size (*n*) for each experimental group/condition, given as a discrete number and unit of measurement
☐	☒	A statement on whether measurements were taken from distinct samples or whether the same sample was measured repeatedly
☐	☒	The statistical test(s) used AND whether they are one- or two-sided<br>*Only common tests should be described solely by name; describe more complex techniques in the Methods section.*
☐	☒	A description of all covariates tested
☐	☒	A description of any assumptions or corrections, such as tests of normality and adjustment for multiple comparisons
☐	☒	A full description of the statistical parameters including central tendency (e.g. means) or other basic estimates (e.g. regression coefficient) AND variation (e.g. standard deviation) or associated estimates of uncertainty (e.g. confidence intervals)
☐	☒	For null hypothesis testing, the test statistic (e.g. *F*, *t*, *r*) with confidence intervals, effect sizes, degrees of freedom and *P* value noted<br>*Give P values as exact values whenever suitable.*
☒	☐	For Bayesian analysis, information on the choice of priors and Markov chain Monte Carlo settings
☒	☐	For hierarchical and complex designs, identification of the appropriate level for tests and full reporting of outcomes
☐	☒	Estimates of effect sizes (e.g. Cohen's *d*, Pearson's *r*), indicating how they were calculated

Our web collection on statistics for biologists contains articles on many of the points above.

Software and code

Policy information about availability of computer code

Data collection	DNA purification from whole blood was done by QIAamp DNA blood kits (Qiagen Benelux BV, Venlo, the Netherlands). The DNA concentration was measured using a NanoDrop spectrophotometer at 260 nm. High-quality DNA was used for genome-wide DNA methylation profile by either Infinium© MethylationEPIC array (BCG and BCG booster) (~850,000 CpG sites) or MethylationEPIC v2 array (500FG) (~937,055 CpG sites).
Data analysis	DNA methylation data was analyzed using minif package in R (v.4.2.0). Epigenome-wide association analysis of urate was performed using a robust linear regression model and its code is freely available at https://github.com/xiaoli-web/Urate

For manuscripts utilizing custom algorithms or software that are central to the research but not yet described in published literature, software must be made available to editors and reviewers. We strongly encourage code deposition in a community repository (e.g. GitHub). See the Nature Portfolio guidelines for submitting code & software for further information.

Data

Policy information about availability of data

All manuscripts must include a data availability statement. This statement should provide the following information, where applicable:

- Accession codes, unique identifiers, or web links for publicly available datasets
- A description of any restrictions on data availability
- For clinical datasets or third party data, please ensure that the statement adheres to our policy

Methylation data can be accessed via EGAS00001007498. Code was provided under https://github.com/xiaoli-web/Urate.

Research involving human participants, their data, or biological material

Policy information about studies with human participants or human data. See also policy information about sex, gender (identity/presentation), and sexual orientation and race, ethnicity and racism.

Reporting on sex and gender	Sex was considered in the study design. The sex of participants was determined by both self-report and molecular-based estimation (estimate using DNA methylation data by minfi getSex function)<br>The sex distribution and the sample size of each sex group were provided in the main text of the paper (result and method sections)<br>The results from the sex-based analysis (both sex-stratified and sex-interaction analyses) were provided.
Reporting on race, ethnicity, or other socially relevant groupings	The discovrty cohort used in this study (300BCG cohort) is western descent population-based cohort. all of these individuals are Dutch healthy individuals.
Population characteristics	The 300BCG cohort is a healthy western descent population-based cohort from the Human Functional Genomics Project. Volunteers were recruited between April 2018 and June 2018 in the Radboud University Medical Center as described previously. All volunteers received a standard dose of 0.1 mL BCG (Intervax, Canada, strain BCG-Bulgaria) intradermally in the left upper arm by a medical doctor. This study was approved by the Ethical Committee of Radboud University Medical Center (NL58553.091.16). There are 184 females and 141 males in this cohort. The average age of males is 27(± 12 SD), and the average age of females is 25(± 9 SD). The BMI and smoking status of these participants were provided in Table S1.
Recruitment	Regarding recruitment of the participants. The exclusion criteria include: acute or chronic illness at the time of sampling; a medical history of immunodeficiency; any febrile illness within 4 weeks before participation; previous BCG vaccination or having lived in tuberculosis endemic countries; history of tuberculosis; any vaccination 3 months before participation; use of systemic medication other than oral contraceptives or acetaminophen; use of antibiotics 3 months before inclusion.
Ethics oversight	This study was approved by the Ethical Committee of Radboud University Medical Center (NL58553.091.16). All participants gave written informed consent.

Note that full information on the approval of the study protocol must also be provided in the manuscript.

Field-specific reporting

Please select the one below that is the best fit for your research. If you are not sure, read the appropriate sections before making your selection.

☒ Life sciences ☐ Behavioural & social sciences ☐ Ecological, evolutionary & environmental sciences

For a reference copy of the document with all sections, see nature.com/documents/nr-reporting-summary-flat.pdf

Life sciences study design

All studies must disclose on these points even when the disclosure is negative.

Sample size	Study design of the 300BCG cohort has been previously published in Koeken VACM, et al, 2020 and 2022. Sample size in this study is based on complete data availability from both DNA methylation and urate.<br><br>Koeken VACM, Qi C, Mourits VP, De Bree LCJ, Moorlag SJCFM, Sonawane V, et al. Plasma metabolome predicts trained immunity responses after antituberculosis BCG vaccination. Locasale JW, editor. PLOS Biol. 2022 Sep 12;20(9):e3001765.<br><br>Koeken VACM, De Bree LCJ, Mourits VP, Moorlag SJCFM, Walk J, Cirovic B, et al. BCG vaccination in humans inhibits systemic inflammation in a sex-dependent manner. J Clin Invest. 2020 Sep 21;130(10):5591–602.
Data exclusions	For methylation data, We excluded poor quality and sex-mismatched samples.

Replication	The identified urate-associated CpG sites were replicated in three additional replication cohorts. Details of these cohorts are provided in the Methods section.
Randomization	Sampe plates are fully randomized to minimize potential confounding by batch effects.
Blinding	No blinding was applied as this study was performed on an observational human study.

Reporting for specific materials, systems and methods

We require information from authors about some types of materials, experimental systems and methods used in many studies. Here, indicate whether each material, system or method listed is relevant to your study. If you are not sure if a list item applies to your research, read the appropriate section before selecting a response.

Materials & experimental systems

n/a	Involved in the study
☒	☐ Antibodies
☒	☐ Eukaryotic cell lines
☒	☐ Palaeontology and archaeology
☒	☐ Animals and other organisms
☒	☐ Clinical data
☒	☐ Dual use research of concern
☒	☐ Plants

Methods

n/a	Involved in the study
☒	☐ ChIP-seq
☒	☐ Flow cytometry
☒	☐ MRI-based neuroimaging

Plants

Seed stocks	NA
Novel plant genotypes	NA
Authentication	NA
